# *In-Situ* Helium Implantation and TEM Investigation of Radiation Tolerance to Helium Bubble Damage in Equiaxed Nanocrystalline Tungsten and Ultrafine Tungsten-TiC Alloy

**DOI:** 10.3390/ma13030794

**Published:** 2020-02-10

**Authors:** Osman El Atwani, Kaan Unal, William Streit Cunningham, Saryu Fensin, Jonathan Hinks, Graeme Greaves, Stuart Maloy

**Affiliations:** 1 Materials Science and Technology Division, Los Alamos National Laboratory, Los Alamos, NM 87545, USA; kunal@lanl.gov (K.U.); saryuj@lanl.gov (S.F.); maloy@lanl.gov (S.M.); 2 Department of Materials Science and Chemical Engineering, Stony Brook University, Stony Brook, NY 11790, USA; william.cunningham@stonybrook.edu; 3 School of Computing and Engineering, University of Huddersfield, Huddersfield HD1 3DH, UK; j.a.hinks@hud.ac.uk (J.H.); g.greaves@huc.ac.uk (G.G.)

**Keywords:** nanocrystalline tungsten, alloy, in-situ electron microscopy, helium bubbles, radiation tolerance

## Abstract

The use of ultrafine and nanocrystalline materials is a proposed pathway to mitigate irradiation damage in nuclear fusion components. Here, we examine the radiation tolerance of helium bubble formation in 85 nm (average grain size) nanocrystalline-equiaxed-grained tungsten and an ultrafine tungsten-TiC alloy under extreme low energy helium implantation at 1223 K via in-situ transmission electron microscope (TEM). Helium bubble damage evolution in terms of number density, size, and total volume contribution to grain matrices has been determined as a function of He^+^ implantation fluence. The outputs were compared to previously published results on severe plastically deformed (SPD) tungsten implanted under the same conditions. Large helium bubbles were formed on the grain boundaries and helium bubble damage evolution profiles are shown to differ among the different materials with less overall damage in the nanocrystalline tungsten. Compared to previous works, the results in this work indicate that the nanocrystalline tungsten should possess a fuzz formation threshold more than one order of magnitude higher than coarse-grained tungsten.

## 1. Introduction

Fusion energy applications require materials to possess superior properties able to withstand extreme environments of high thermal loads, transient heat fluxes, high fluxes of helium (He) plasma particles, and fast neutrons [1]. Furthermore, neutron irradiation can also lead to solid transmutation products and He gas formation [2]. These challenging conditions can exacerbate damage in materials facing the plasma. Tungsten is currently one of the best candidates for the divertor armor and is to be used in ITER (originally the International Thermonuclear Experimental Reactor), but has demonstrated several drawbacks (in terms of microstructural and mechanical property changes) when exposed to extreme irradiation conditions [3,4,5]. One of the most studied microstructural changes is the formation of fuzz: a high density of dendritic structures that can develop on the surface of tungsten at high temperatures and He plasma fluxes [3]. While the precise mechanisms of fuzz formation are still under investigation, it is understood to occur due to a high density of He bubbles forming near the tungsten surface [6,7]. Several routes have been suggested in the quest to design irradiation resistant materials capable of mitigating microstructural changes and fuzz formation in a plasma facing material. Refining the grain size to the nanocrystalline regime, and thereby increasing the density of interfaces that act as defect sinks, has been proposed as a method to mitigate these changes [8,9]. Particle reinforcement, where the particles act as defect annihilation sites, is also a suggested route to increase the irradiation resistance of the material and enhance its mechanical properties and thermal stability [10,11,12]. The hypothesis is that materials that are radiation tolerant to bubble formation should lead to an increased He^+^ fluence threshold for fuzz formation (at fuzz formation conditions) if fuzz formation depends on the same conditions as proposed in the literature [6]. El Atwani et al. studied nanocrystalline tungsten with a 35 nm average grain size [13] and an ultrafine tungsten alloy with TiC dispersoids (W-TiC 1.1%) [14] under heavy ion irradiation (to mimic fast neutron damage). Both types of materials have shown enhanced irradiation tolerance when compared to commercially-available tungsten. Damage due to loop formation was shown to be minimal in the nanocrystalline tungsten and decreased with time (due to loop annihilation and dissolution under heavy ion cascades) in the W-TiC (1.1%). The response of these materials, however, due to low energy He^+^ implantation is still unknown. The testing of severe plastically deformed (SPD) nanocrystalline and ultrafine tungsten, where both elongated ultrafine (˂500 nm) and nanocrystalline (˂100 nm) grains coexist under low energy He^+^ implantation and high temperatures (1223 K), showed a trend in both damage and He bubble formation as a function of grain size [15]. Grains of less than 60 nm (in elongated grains, size was defined to be the distance across the middle in the narrower direction) demonstrated a high irradiation tolerance to bubble formation and grains of ~35 nm were shown to possess minimum damage.

Here, we present a detailed study investigating the radiation tolerance to bubble formation under low energy (2 keV). Implantation of He^+^ at a high temperature (1223 K) (similar conditions to previous SPD work [15]) of the equiaxial nanocrystalline tungsten with an average grain-size 85 nm (referred to as NCW herein) was formed by magnetron deposition, and the tungsten-TiC (1.1%) (labelled as W-TiC (1.1%)) formed by hot iso-static pressing of tungsten powders with TiC dispersoids. The experiments were performed in-situ within the transmission electron microscope (TEM) in the MIAMI-2 system (Microscope and Ion Accelerators for Materials Investigations) at the University of Huddersfield [16]. Bubble formation, distribution, and evolution in the materials were studied by quantifying bubble density, average size, and total change in volume (in the grain matrices of the material due to bubble formation) as a function of implantation He^+^ fluence. Together with the previous work in SPD tungsten, the results constitute a comparable set of bubble/damage evolution profiles and distribution for different ultrafine and nanocrystalline grades (pure and alloyed) that should assist in the understanding of nanocrystalline and ultrafine material behavior under He^+^ implantation and the design of materials with higher irradiation resistance for fusion applications.

## 2. Materials and Methods

**Figure 1:**
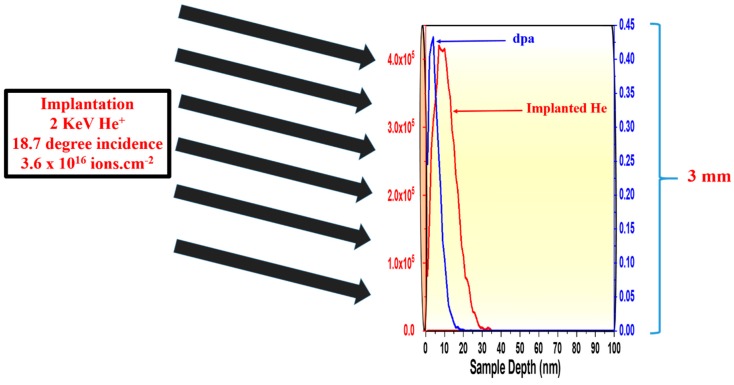
A schematic showing the sample shape, implanted He (red curve), and displacement per atom (dpa) damage (blue curve) distributions of 2 keV He^+^ (as determined by SRIM). The thickness of the sample is magnified for the purpose of overlapping the implanted He and displacement damage distributions. (For interpretation of the references to color in this figure legend, the reader is referred to the web version of this article.)

The TEM sample (~100 nm thickness) preparation methodology and detailed morphology of the samples prior to implantation have been described in detail previously [13,14]. The implantations were incident at 18.7° from the surface normal with fluxes of 8.8 × 10^13^ and 6.8 × 10^13^ ion.cm^−2^.s^−1^ for the NCW and W-TiC (1.1%), respectively, to a total He^+^ fluence of 3.6 × 10^16^ ion.cm^−2^. Displacement damage and He distributions were found by the Kinchin-Pease model in the Stopping Range of Ions in Matter (SRIM) Monte Carlo computer code (version 2013) [17], using 70 eV as a displacement threshold [18]. He bubbles were characterized using bright-field TEM imaging at under-focused (bubbles appear bright) conditions [19]. For every He^+^ fluence reported, different grains (about 7 grains in the W-TiC (1.1%) sample and 15 grains in the NCW sample) in every sample were quantified at different He^+^ fluences. In every grain, several small circles (3–8 depending on the grain size) of the same area were drawn randomly and the number of He bubbles and their corresponding sizes were found. Averages were calculated from the results in all grains. A detailed illustration of the quantification process was published in the supplemental of reference [20]. Figure 1 shows a schematic diagram of the sample, implantation conditions and overlapping ion and displacement damage distributions. The projected He peak is ~12–15 nm, which is much less than the nominal thickness of the film (~100 nm). This is an important consideration when comparing and illustrating phenomena (such as bubble formation and defect annihilation/recombination) that can be affected by the proximity to free surfaces. However, surface proximity effects becomes negligible in nanocrystalline and ultrafine grains when the grain boundary to surface ratio approaches a value of 1 [21]. The grain boundary to surface ratios on the equiaxed 85 nm tungsten and the W-TiC (1.1%) were measured taking into consideration the upper and the bottom surface of the foils and assuming edge-on grain boundaries, and were found to be 3.8 and 0.3, respectively. Interfaces of TiC particles and the tungsten matrix inside the grains were not considered in the case of the W-TiC (1.1%) grade. These values are relatively high compared to fine- or coarse-grained grades. For example, a corresponding grain boundary to surface ratio for a fine-grained tungsten grade with an average grain size of ~2 µm would be ~0.04 [13]. Due to the shallow depth of implanted He in this work, no considerable variations in proximity effects are expected between the two grades investigated.

## 3. Results and Discussion

### 3.1. He Bubble Formation and Growth

**Figure 2:**
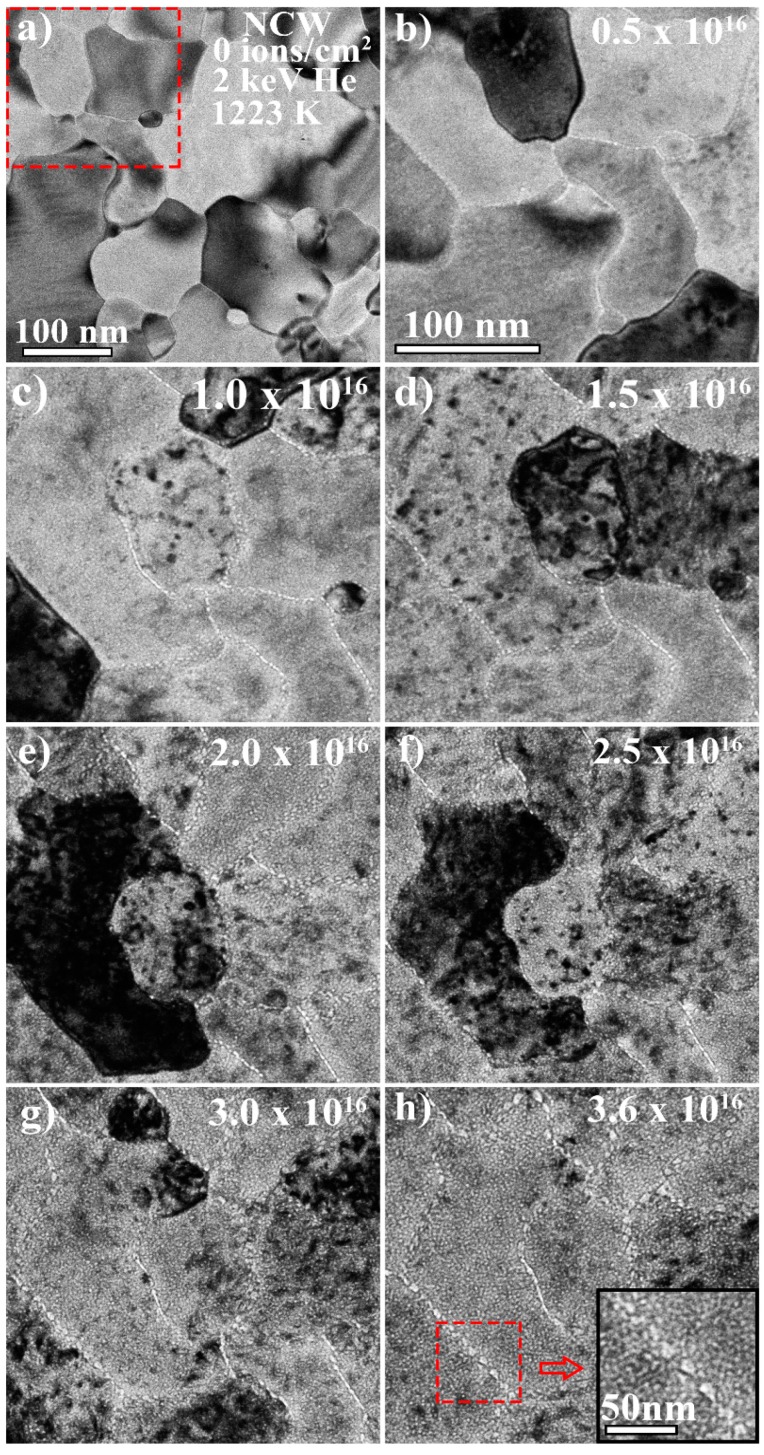
(**a**–**h**): Bright-field TEM micrographs of a small implanted region taken under Fresnel conditions (under-focused) showing He bubble formation and evolution as a function of He^+^ fluence in the grain matrices and grain boundaries in equiaxial nanocrystalline tungsten (NCW) with an average grainsize of 85 nm implanted in-situ with 2 keV He^+^ at 1223 K. Scale bar of (**b**–**h**) is the same and is shown in (**b**). Red box in (**a**) approximately represents a magnified region presented in (**b**) to (**h**).

**Figure 3:**
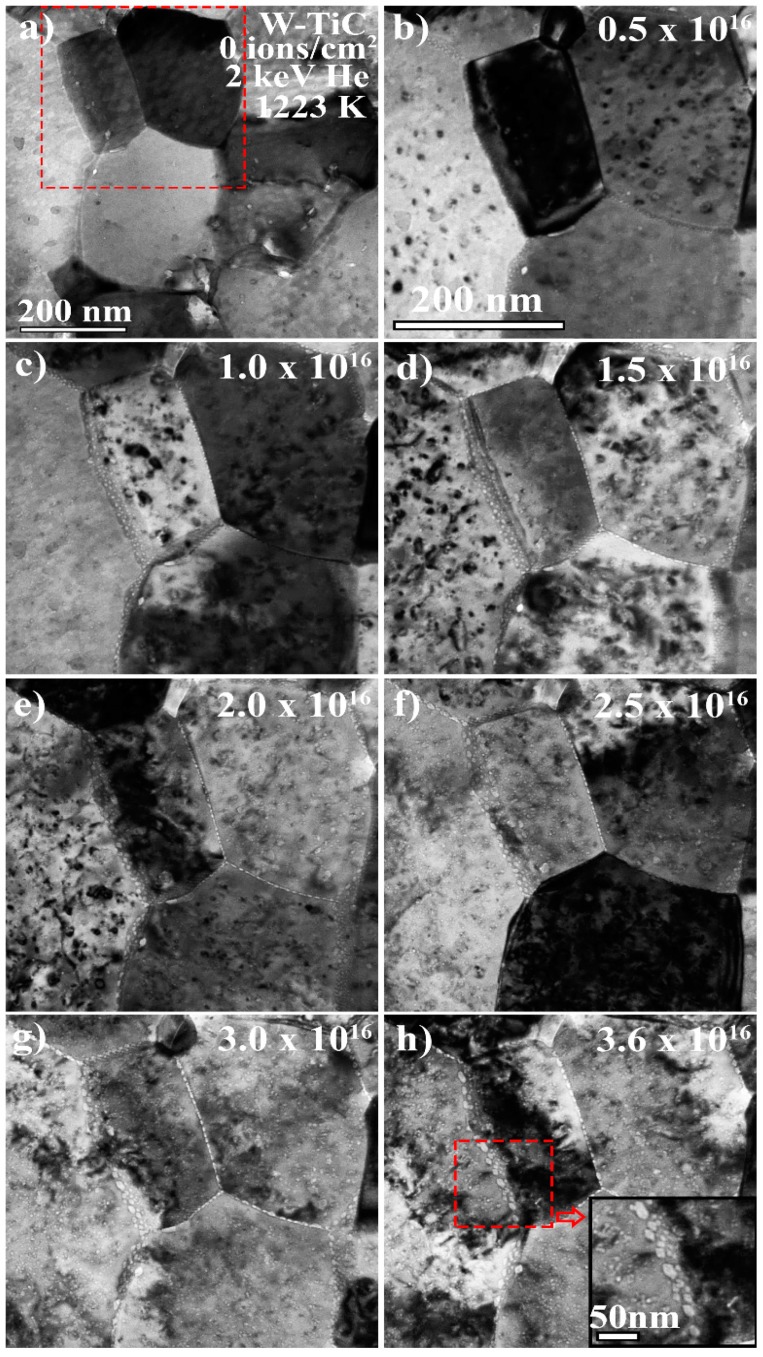
(**a**–**h**): Bright-field TEM micrographs of a small implanted region taken under Fresnel conditions (under-focused) showing He bubble formation and evolution as a function of He^+^ fluence in the grain matrices and grain boundaries in W-TiC (1.1%) implanted in-situ with 2 keV He^+^ at 1223 K. Scale bar of (**b**–**h**) is the same and is shown in (**b**). Red box in (**a**) approximately represents a magnified region presented in (**b**) to (**h**).

At the temperature studied in this work (1223 K), vacancies, interstitials, and small He–vacancy complexes are mobile and hence He bubbles are expected to form [22]. The He bubble density, average size, and the consequential change in volume in the grain matrices due to He bubble formation, as a function of He^+^ fluence, not only reveal the total damage (as defined here) in the sample but also demonstrate the damage evolution profiles and elucidate the variations in the response of the two materials. Figure 2 and Figure 3 show bright-field TEM images taken under Fresnel conditions (under-focused conditions where bubbles appear bright) of the NCW and W-TiC (1.1%) as a function of He^+^ implantation fluence and indicate He bubble formation. In both cases (Figure 2b and Figure 3b), He bubble formation was more evident at the grain boundaries than in the bulk grain matrices. As the fluence was increased, grain boundaries demonstrated larger He bubble formation than that in the grain matrices. While this demonstrates efficient He trapping by the grain boundaries, the mechanisms involved in this process can be complicated [23]. At the He^+^ energy in this work (2 keV), the number of vacancies generated per ion is 0.5 for a 70 eV displacement threshold [18] according to SRIM calculations. Therefore, He_n_V_m_ complexes of n/m larger than 1 are expected to dominate especially taking the inevitable dynamic annealing of vacancies into account. Such complexes have very large migration energies and are not expected to migrate [24]. Only complexes with migration energies close to that of vacancies (1.7 eV) [25] can contribute to He bubble formation at the grain boundaries under these conditions and these are mainly complexes with a higher vacancy content [24,26]. Interstitial-He trapping by the grain boundaries (2D trapping) can also occur and then He bubbles can form through trap mutation processes as well as via vacancy migration to the grain boundary [27]. The probability of trap mutation and He bubble formation on the grain boundaries can be high at this temperature due to higher gas pressure and the decrease in self-interstitial formation energy [28]. Such mechanisms are therefore expected to contribute to the observed formation of larger He bubbles at the grain boundaries; nevertheless, understanding large He bubble growth on grain boundaries requires further coordinated and complimentary experimental and modelling work.

### 3.2. He Bubble Evolution as a Function of He^+^ Fluence

**Figure 4:**
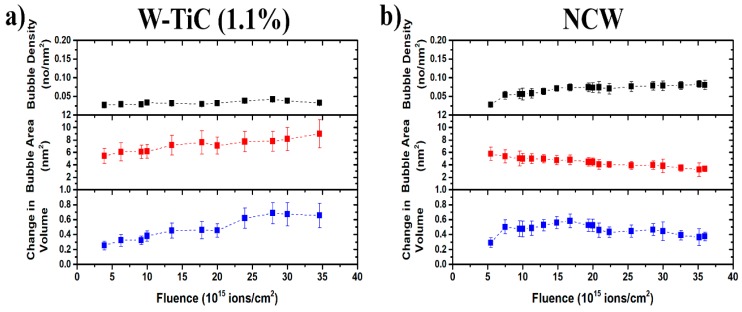
(Color online) Helium bubble density, average area, and the total change in volume in the grain matrices of (**a**) W-TiC and (**b**) NCW as a function of He^+^ implantation fluence. (For interpretation of the references to color in this figure legend, the reader is referred to the web version of this article.)

He bubble damage evolution in the grain matrices, as quantified by changes in the bubble number density, area, and the total change in volume (found using Δvv $=\frac{4}{3}\pi \;r_{c}^{3}N_{v}$ where $N_{v}$ is the bubble density in a 100 nm thick foils and $r_{c}$ is the average radius of the bubbles) due to bubble formation in the grain matrices for both tungsten grades, are shown in Figure 4 (data adjusted for the NCW for variations in focus, in different video segments, occurring during the dynamic in-situ experiments, as described in Figure S1 and the Supplemental Materials). The He bubble density in the NCW grade increased with He^+^ fluence and reached a plateau (at a density of ~ 0.125 nm^−2^), while the average bubble size showed a decrease as a function of He^+^ fluence and reached an average size of 3 nm^2^. The corresponding change in volume showed an increase with He^+^ fluence at the start of implantation, peaked at ~0.7, and then decreased to ~0.4 at the last He^+^ fluence (3.5 × 10^16^ ion.cm^−2^). In the case of W-TiC (1.1%), the number density increased first (when the bubbles became visible) to 0.025 nm^−2^ and then saturated throughout the implantation, while the average bubble area increased as a function of time (up to 8 nm^2^) in a similar trend to the corresponding change in volume, which reached a value of 0.6.

### 3.3. Damage Evolution Comparison

While the final total change in volume in the NCW is about half the change in volume in the W-TiC, indicating higher radiation tolerance of NCW to He^+^ implantation, the damage evolution (and thus, the bubble density profile) is quite different. In the W-TiC, the change in volume followed the average bubble area profile (whereas the bubble number density was saturated). Therefore, bubble damage in this case is due to the growth of existing He bubbles through the absorption of vacancy, He, and/or He–vacancy complexes. The small bubble density in W-TiC compared to the NCW bubble density, in this case, indicates greater defect accumulation (vacancies and possible He–vacancy complexes with low migration energies) of the former. In addition to the role of high densities of grain boundaries in limiting irradiation or implantation damage, TiC dispersoids have been shown to assist in the defect recombination and absorption of excess vacancies acting as vacancy-biased sinks and to contribute to a higher radiation tolerance of the grain matrices to irradiation damage [12,14]. The same W-TiC material has previously been demonstrated, under heavy ion irradiation, to have higher irradiation resistance to cavity and loop formation (in the grain matrices) compared to commercial coarse-grained tungsten [14]. In previous work [15], nanocrystalline and ultrafine tungsten (where both elongated nanocrystalline and ultrafine grains coexist—SPD material) was implanted under the same conditions used in the current work. Bubble density in the grain matrices of the W-TiC was five times higher than in the SPD material but the average bubble area was ~2.5 times lower in the former. However, the total change in volume was the same. Relatively lower defect mobility, due to the impurities in the W-TiC, could potentially lead to more bubble nucleation sites compared to pure tungsten.

**Figure 5:**
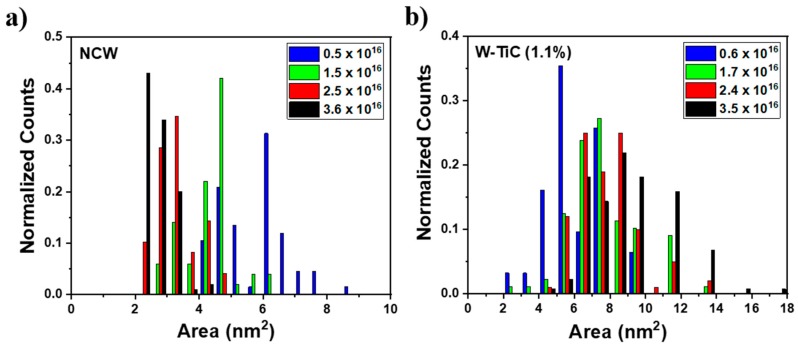
(Color online) normalized bar graphs of bubble size distributions in the grain matrices in (**a**) NCW and (**b**) W-TiC as a function of implantation He^+^ fluence. (For interpretation of the references to color in this figure legend, the reader is referred to the web version of this article.)

The NCW demonstrated a high density of very small bubbles in the grain matrices which was unexpected. It also showed a decrease in the average bubble size as a function of He^+^ fluence. The decrease in average size could be due to the nucleation of new small bubbles, which can decrease the average bubble size. However, from the histograms of bubble sizes at four different He^+^ fluences shown in Figure 5, the average bubble size decrease was shown to be due to a decrease in the sizes of the individual bubbles. Moreover, the average bubble size continued to decrease even after the bubble density plateaued. Such a decrease in bubble size has to be associated with the balance of interstitial and vacancy fluxes against the bubbles in addition to any possible thermal emissions of vacancies at 1223 K (the latter can be difficult due to the high binding energies of vacancies and He) [24] and internal pressure changes due to the He concentration change in the bubble. Both a higher level of interstitial absorption and thermal vacancy emission (the latter of which being suppressed as mentioned above) can lead to the shrinkage of the bubbles. Moreover, nanoporosity from magnetron deposited films can be responsible for higher densities of bubble nucleation sites but this porosity can reduce with irradiation [29] due to adatom accumulation occurring before reaching a stable bubble size. Nanoporous materials were shown to enhance irradiation tolerance in different materials [30,31,32]. Nanoporosity and possible impurities can also limit the mean free path of defect mobility and thus explain the high density of He bubbles in the NCW. Dislocation loops formed in the NCW were shown to be highly mobile [13]. The interaction of these loops with He bubbles may cause them to shrink. There can also be an effect of residual stress on defect absorption, which is yet to be understood in nanocrystalline materials. Under heavy ion irradiation with no gas introduction, the NCW grades demonstrated higher irradiation resistance to void formation (but with higher void density) compared to the SPD material and commercial coarse-grained tungsten [13]. It was shown in the same study that interstitial and vacancy defects have a higher recombination probability at smaller grain sizes, meaning that NCW would have less cavity damage. In the current work, the bubble sizes at the grain boundaries in the NCW (~8–10 nm as shown in inset in Figure 2h) were smaller than those in the W-TiC grade (~20–25 nm as shown in the inset in Figure 3h). They were also smaller than the bubbles (~10 nm) at the grain boundaries in the SPD material at the same He^+^ fluence [15]. This represents lower overall bubble damage, which is indicative of a lower He–vacancy complex (with low migration energies) and vacancy transport to the grain boundaries (presumably due to higher levels of recombination). Moreover, the high density of grain boundaries can lead to lower defect transport (to grain boundaries) per boundary. It should be mentioned that the very large bubble sizes at the grain boundaries in the W-TiC material could be due to the TiC impurities at the grain boundaries, which can change the grain boundary sink efficiency and have been shown to be vacancy-biased, as previously discussed. The SPD material in reference [33] showed one order of magnitude higher He^+^ fluence threshold for fuzz formation compared to commercial tungsten. The NCW in the current work (with its lower total bubble damage in the grain matrices and grain boundaries) is similarly expected to demonstrate an improved fuzz resistance. Such a material needs to be examined under plasma conditions to assess its mechanical property behavior to evaluate its suitability to fusion conditions.

## 4. Conclusions

In summary, He bubble damage evolution was studied using in-situ TEM as a function of He^+^ implantation fluence for two strong fusion material candidates (NCW and ultrafine W-TiC alloy). The results have been compared to previously studied SPD nanocrystalline and ultrafine tungsten [15]. Grain boundaries were shown to be decorated with large bubbles, indicating efficient He trapping. He bubble evolution, profiles, and ov]erall bubble damage (total change in volume in grain matrices and grain boundaries) were found to be different in both grades. The NCW showed a higher density of small bubbles but a lower overall change in volume than the W-TiC grade and the previously published SPD tungsten. The high density of small bubbles in the NCW was unexpected but several phenomena can govern this observation. Since SPD tungsten possessed a He^+^ fluence threshold for fuzz formation that was one of order of magnitude higher than coarse-grained tungsten, the NCW is then expected to have an even higher He^+^ fluence threshold (for fuzz formation). This is true if the fuzz formation mechanism depends on high bubble densities near the surface, as illustrated in previous literature works. These results indicate that while these materials may have improved fuzz resistance under plasma exposures and an enhanced tolerance to He damage due to neutron transmutation reactions, further studies of these materials under fusion conditions need to be performed to develop materials that can withstand these extreme conditions.

## Supplementary Materials

The following are available online at https://www.mdpi.com/1996-1944/13/3/794/s1, Figure S1: (Color online) Helium bubble density, average size, and total change in volume in the grain matrices of NCW and W-TiC as functions of He^+^ fluence. (a) shows the original and the adjustment data.
