# Supplementary material for: In-Situ Helium Implantation and TEM Investigation of Radiation Tolerance to Helium Bubble Damage in Equiaxed Nanocrystalline Tungsten and Ultrafine Tungsten-TiC Alloy
Source: Materials (Basel). 2020 Feb 10;13(3):794. doi: 10.3390/ma13030794 (PMC7040824; doi:10.3390/ma13030794)
Supplement: Supplementary file 1 [file materials-13-00794-s001.pdf]

materials

Supplementary Material

In-situ Helium Implantation and TEM Investigation of Radiation Tolerance to Helium Bubble Damage in Equiaxed Nanocrystalline Tungsten and Ultrafine Tungsten-TiC Alloy

Osman El Atwani *, Kaan Unal, William Streit Cunningham, Saryu Fensin, Jonathan Hinks, Graeme Greaves and Stuart Maloy

NCW video data adjustment

In the NCW, adjusted data curves are plotted in addition to the original generated data. The original data indicates steps at 1×10^{15} ion.cm^{-2} and 1×10^{16} ion.cm^{-2} due to a change in focus conditions from one video segment to another. These were adjusted by considering the last value (i.e., density or area) in the previous video segment with the first value from the following video segment (which should match since they correspond to the same fluence). The original and adjusted data are shown in the Figure S1.

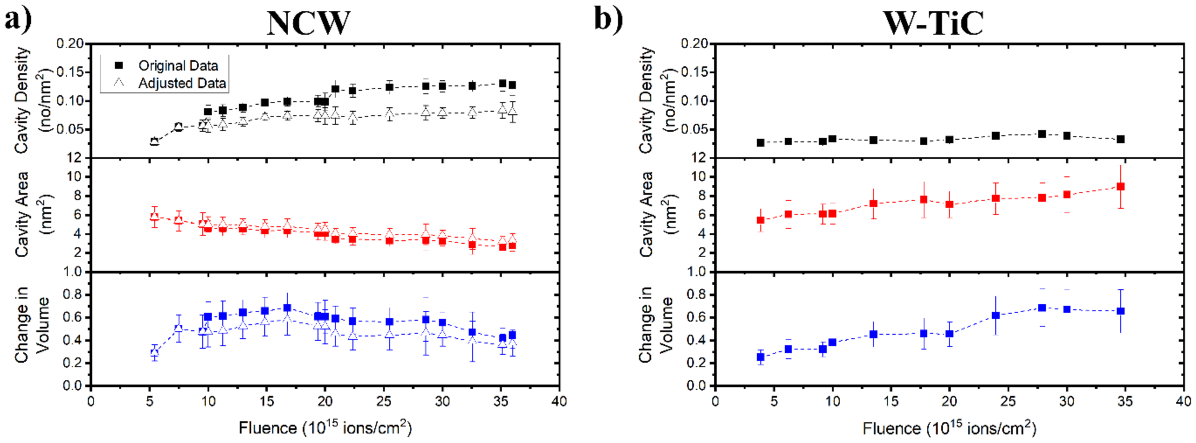

Figure S1. (Color online) helium bubble density, average size, and total change in volume in the grain matrices of NCW and W-TiC as functions of fluence. **a**) shows the original and the adjustment data.
